# Microfabrication of Nonplanar Polymeric Microfluidics

**DOI:** 10.3390/mi9100491

**Published:** 2018-09-25

**Authors:** Pin-Chuan Chen, Chung-Ying Lee, Lynh Huyen Duong

**Affiliations:** Department of Mechanical Engineering, National Taiwan University of Science and Technology, Taipei 10607, Taiwan; m10403114@mail.ntust.edu.tw (C.-Y.L.); duonghuyenlynh@gmail.com (L.H.D.)

**Keywords:** nonplanar microfluidic chip, micromachining microfluidics, microfluidics

## Abstract

For four decades, microfluidics technology has been used in exciting, state-of-the-art applications. This paper reports on a novel fabrication approach in which micromachining is used to create nonplanar, three-dimensional microfluidic chips for experiments. Several parameters of micromachining were examined to enhance the smoothness and definition of surface contours in the nonplanar poly(methyl methacrylate) (PMMA) mold inserts. A nonplanar PMMA/PMMA chip and a nonplanar polydimethylsiloxane (PDMS)/PMMA chip were fabricated to demonstrate the efficacy of the proposed approach. In the first case, a S-shape microchannel was fabricated on the nonplanar PMMA substrate and sealed with another nonplanar PMMA via solvent bonding. In the second case, a PDMS membrane was casted from two nonplanar PMMA substrates and bonded on hemispherical PMMA substrate via solvent bonding for use as a microlens array (MLAs). These examples demonstrate the effectiveness of micromachining in the fabrication of nonplanar microfluidic chips directly on a polymeric substrate, as well as in the manufacture of nonplanar mold inserts for use in creating PDMS/PMMA microfluidic chips. This technique facilitates the creation of nonplanar microfluidic chips for applications requiring a three-dimensional space for in vitro characterization.

## 1. Introduction

Microfluidic technology has been steadily progressing since micro-gas chromatograph was first reported in 1979 [1]. In the 1970s and 1980s, silicon and glass substrates were mainstream substrate materials due to the fact that lithography was the primary microfabrication method during that period. Initially, the expense and level of skill required for microfabrication hindered the expansion of microfluidics into other fields. The soft lithography technique reported in [2] greatly facilitated the fabrication of microfluidic chips, thereby prompting researchers from a variety of backgrounds to use microfluidics in interesting new applications [3]. The commercialization of microfluidics products has shifted the focus to affordability and disposability, particularly in point-of-care (POC) systems. The advent of polymer substrates at the end of the 1990s opened the door to low-cost mass production, transparent devices, and surface-modifiability [4]. Numerous research groups have developed polymeric microfluidic chips for conducting experiments [5,6,7]. Another low-cost microfluidic platform was developed using a paper substrate [8,9], which foregoes the need for an external driving force.

Lithography is used in the manufacture of planar structures on flat substrates. Advancements in this field led to the development of spin-coating processes to apply photoresist with uniform thickness and ensure alignment between the optical mask and photoresist. Planar microfluidics is a major platform used to study a variety of chemical/bio applications; however, the two-dimensional structure of these devices is ill-suited to the study of many interesting topics, such as cell cultures and tissue engineering for in vitro characterization. Edmonson et al. [10] described how cells in a three-dimensional (3D) culture environment can differ morphologically and physiologically from cells in a 2D culture environment. This fact can alter the cellular response to drugs, and has caused the failure of numerous clinical trials. Baker et al. [11] demonstrated how a 3D microenvironment could be used to study the fundamental features of 3D cell cultures, such as the means by which the structure, composition, and 3D distribution of adhesive domains can alter cell shape and cytoskeletal organization, with subsequent effects on cell signaling and function. A microscale 3D environment is essential to truly mimic the in vivo environment for its use in characterizing cell responses in the fields of drug discovery, cell biology, stem cell research, and many other cell-based areas of investigation.

A number of 3D microfluidic devices have been developed; however, most of them are based on planar fabrication processes. Do et al. [12] presented a maskless direct writing technique for 3D microfluidic chips, in which thermoplastic substrates with patterns fabricated using a cutter plotter undergo thermal bonding to create a multilayered sealed microfluidic chip for experiments. Zhang et al. [13] reported an add-on process similar to stamp-stick bonding [14] to create 3D polymeric microfluidic chips. In that study, each PDMS layer was created and bonded one by one. 3D microfluidic chips have not been limited to polymeric substrates. Martinez et al. [9] reported a 3D microfluidic paper-based analytical device (µPAD) on paper substrates using double-sided adhesive tape for detecting glucose levels and proteins. Additive manufacturing (i.e., 3D printing) has also been used to create 3D microfluidic chips for a variety of microfluidic devices. Gong et al. [15] used a stereolithographic (digital light processing (DLP)-stereolithography (SLA)) 3D printer to fabricate a microfluidic chip with a cross-section of only 18 × 20 μm. In that study, they introduced a mathematical model to assist in the selection of appropriate resins for high-resolution printing. Those advances enabled them to fabricate 3D serpentine structures and microchannels with a high aspect ratio. Chan et al. [16] reported a one-step molding process for the fabrication of 3D microfluidic networks, wherein 3D printing was used to fabricate mold inserts followed by PDMS casting. They presented several interesting examples, including a basket-weaving network, a 3D chaotic advective mixer, microfluidic peristaltic valves, and an “injection-on-demand” microfluidic device.

3D printing has been used to create 3D microfluidic chips directly; moreover, 3D printing with replication technique were used to fabricate 3D microfluidic chips on PDMS substrate. Glick et al. [17] used 3D printing to create nonplanar mold inserts for use in PDMS casting to create 3D, nonplanar microfluidic devices. They addressed double-sided molding, bonding, alignment, and rapid assembly to create single-layer membrane microvalve microfluidics and six-layer structures. Hwang et al. [18] used 3D printing in conjunction with PDMS casting to create complex, nonplanar, arbitrary microchannels. 3D printing is used to create mold inserts with a helical structure, followed by PDMS casting and demolding to create helical microchannels for experiments. Saggiomo et al. [19] reported an interesting method to create a 3D microfluidic chip. They used 3D printing to create a 3D acrylonitrile butadiene styrene (ABS) mold, then PDMS casting was applied, and finally acetone solvent solution was used to dissolve the ABS to create a 3D and transparent microfluidic chip for experiments. Flexible microfluidics has been reported for use as a wearable device [20], but deformation would be observed when attaching a soft and planar microfluidic device to wrists or arm; hence a nonplanar mold insert would be useful in creating a flexible microfluidics without causing deformation and with improving adhesion.

Fabricating microchannels on nonplanar PMMA substrates is very different and far more complex than fabricating microchannels on planar substrates. Hence, our objective in the current study was to use micromachining to create nonplanar microfluidic devices with the aim of gaining insight into the fabrication of 3D microfluidic chips of greater complexity for various applications. Micromilling is a cost-efficient, powerful fabrication tool used to create a variety of polymeric microfluidics. Guckenberger et al. [21] clearly described the pros and cons of using micromilling to create planar polymer microfluidic chips. Creating microfluidic chips via micromilling has a number of advantages over 3D printed chips, such as the ability to create microchannels of smaller dimension with lower surface roughness on transparent materials. We demonstrated the efficacy of the proposed scheme by fabricating two nonplanar microfluidic chips: a nonplanar polymeric microfluidic chip with S-microchannel and a nonplanar PDMS/PMMA microfluidic chip. In the first example, micromilling was used to fabricate an S-microchannel directly on the nonplanar PMMA substrate, whereupon solvent bonding was used to seal the microchannel against another nonplanar substrate. The second example is used as an adjustable PDMS microlens array (MLA) on a hemispheric PMMA substrate. Nonplanar PMMA mold inserts fabricated via micromilling were used in PDMS casting to produce a PDMS membrane that was bonded to a hemispheric PMMA substrate via solvent bonding. These two examples clearly demonstrate the efficacy of using micromilling to create nonplanar PMMA microfluidic chips or PDMS/PMMA microfluidics chips.

## 2. Fabrication of Nonplanar PMMA/PMMA Chips

**Figure 1:**
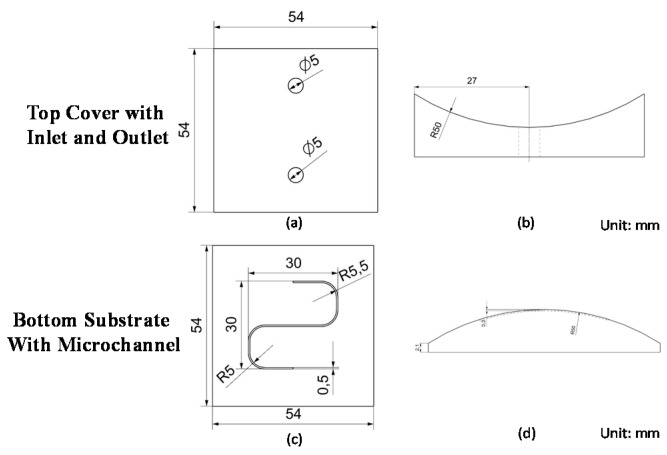
Layout of nonplanar microfluidic chip with S-microchannel: (**a**) design and dimensions of top cover (top view); (**b**) design and dimensions of top cover (side view); (**c**) design and dimensions of bottom substrate (top view); (**d**) design and dimensions of bottom substrate (side view).

**Figure 2:**
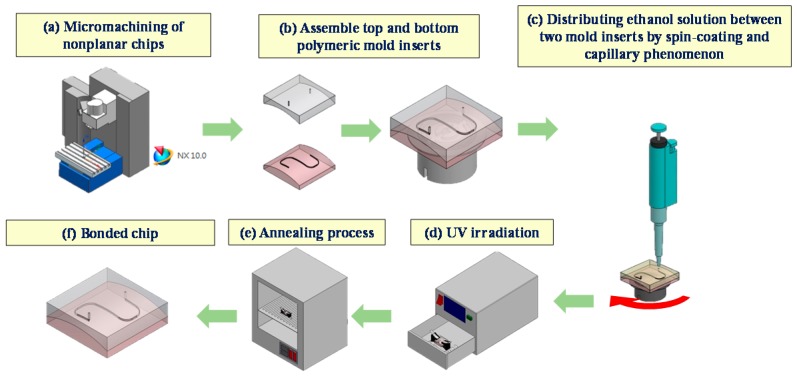
Microfabrication of nonplanar microfluidic chip with S-microchannel: (**a**) micromachining nonplanar substrates; (**b**) fixing two assembled substrates on rotation table; (**c**) injection of ethanol solution into microchannel using pipette followed by spin-coating for uniform distribution between substrates; (**d**) exposure of assembled chip to ultraviolet (UV) irradiation; (**e**) final annealing by oven; (**f**) bonded chip ready for experiments.

An S-microchannel was created in a nonplanar PMMA substrate in order to study the process of fabricating nonplanar polymeric microfluidic chips. Figure 1 illustrates the layout and dimensions of the microfluidic chip with an S-microchannel. Figure 1a,b presents the top cover with through-holes used as inlets and outlets for the transfer of reagents. Figure 1c,d presents the bottom substrate with a micromilled microchannel. The main challenge posed by this fabrication process lies in the use of micromachining to create well-matched machined contours on the top and bottom substrates. A failure in this respect would make it impossible to achieve a satisfactory bond using chemical solvent bonding. Figure 2 illustrates the fabrication process of the microfluidic chip with S-microchannel, including the micromilling process (Figure 2a), solvent bonding (Figure 2b) involving spin-coating (Figure 2c), ultraviolet (UV) exposure (Figure 2d), and annealing (Figure 2e).

### 2.1. Tool Path for Micromachining Nonplanar Substrates

**Figure 3:**
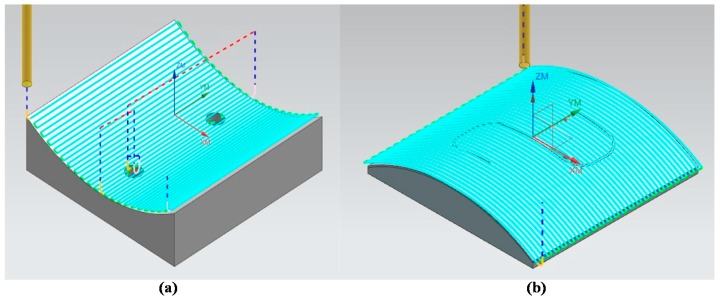
(**a**) Blue lines indicate planned cutting path for top cover substrate; (**b**) blue lines indicate planned cutting path for bottom substrate.

The design of the microfluidic chip with S-microchannel is illustrated in Figure 1. The computer-aided design/computer-aided manufacturing (CAD/CAM) file of the device was imported into NX software (NX 10, Siemens, TX, USA) to set up the cutting path. NX is a powerful CAD/CAM software for machining, with numerous parameters that can be adjusted according to the cutting scenario, including cutting speed, depth per cut, and feed rate. The tolerance and cutting path are also crucial to machining well-defined contours with a smooth surface profile. Tolerances can be lowered to minimize deviations between the designed and machined contours; however, this greatly slows down the machining process. Thus, we conducted a number of preliminary trials to identify the tolerances required to achieve smooth contours on a nonplanar substrate within a reasonable time frame. NX software allows the user to select from among numerous types of cutting path that are suitable for the layout and precision that are required for the finished part. In this study, the smoothness of the curly contour was a particular concern. Thus, we set the path of the cutting tool to proceed from one edge of the substrate to the other edge, rather than planning any turns or stops in the middle of the substrate. Our objective in planning this path was to prevent a discontinuous machined surface resulting from the backlash of ball screws during the machining process. The planned cutting paths are presented in Figure 3. Figure 3a shows the cutting path plan for the top of the PMMA mold insert, and Figure 3b shows the cutting path plan for the bottom of the PMMA mold insert. The blue line on both nonplanar substrates indicates the cutting path from one edge of the substrate to the other.

### 2.2. Micromachining

**Figure 4:**
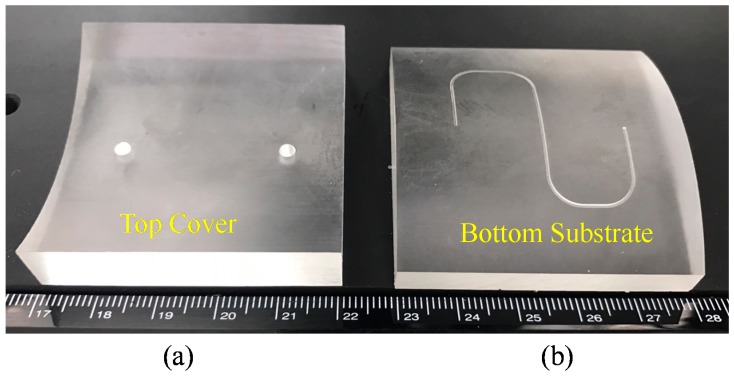
(**a**) Micromachined nonplanar top cover; (**b**) micromachined nonplanar bottom substrate with a S-microchannel.

Micromilling was implemented in three stages with varying degrees of precision: rough, semi-rough, and finish machining. Our objective in using different combinations of cutting parameters was to create smooth nonplanar PMMA mold inserts with minimal residual scallop height. The parameters and cutting tools differed according to the degree of precision. Rough machining using a square endmill was meant to rapidly remove polymeric material, whereas the objectives behind the semi-rough and finish machining (using a ball endmill) was to create a smooth finished surface. Following the fabrication of the free-form PMMA substrate, an S-microchannel was cut into the bottom PMMA substrate. Figure 4 presents the finished nonplanar PMMA mold inserts. Figure 4a shows the top PMMA cover and the Figure 4b shows the bottom PMMA substrate with engraved S-microchannels. The detailed machining process is listed in Tables S1 and S2. The surface roughness of the top and bottom PMMA substrates were listed in the Table S3, in which the average surface roughness of the top PMMA substrate is 1.13 μm while the average surface roughness of the bottom PMMA substrate is 0.9 μm.

### 2.3. Solvent Bonding via Spin-Coating

After fabricating the top cover and bottom substrate, we employed a solvent bonding method similar to that outlined in [22] for the assembly of the two machined PMMA substrates. Immersion and dropping are the most common methods used to distribute ethanol solution on thermoplastic substrates; however, neither of these methods is particularly effective in achieving uniformity, particularly when using solvents that evaporate quickly. In this study, we opted for spin-coating to achieve greater uniformity in the distribution of the ethanol solution. Figure 2b,c illustrate the steps involved in the bonding of the two substrates. After bringing together the two pieces of PMMA substrate (Figure 2b), they were fixed onto the rotation platform of a spin-coating machine (Figure 2b). Ethanol solution was then injected into the microchannel manually using a pipette (Figure 2c) to distribute the ethanol solution within the gap formed between the top and bottom PMMA substrates. The distribution of the solution was facilitated by capillary action and the centrifugal force generated by the spinning movement. The platform was rotated at a speed of 190 rpm for 10 s to ensure the uniform distribution of the ethanol solution. The entire assembly was then immediately placed in an irradiation device under a UV intensity of 80 mW/cm^2^ (Figure 2d) for an exposure duration of 60 s. Following UV irradiation, the bonded thermoplastic chips were subjected to annealing in an oven before undergoing a leakage test and a burst test to determine the quality of the resulting bond. The leakage test involved the injection of red food dye into the microfluidic chip, during which a tool microscope was used to look for any signs of leakage. The burst test involved the assembly of a fluidic system aimed at characterizing the bonding strength.

## 3. Extended Nonplanar Fabrication Process for PDMS/PMMA Chip

**Figure 5:**
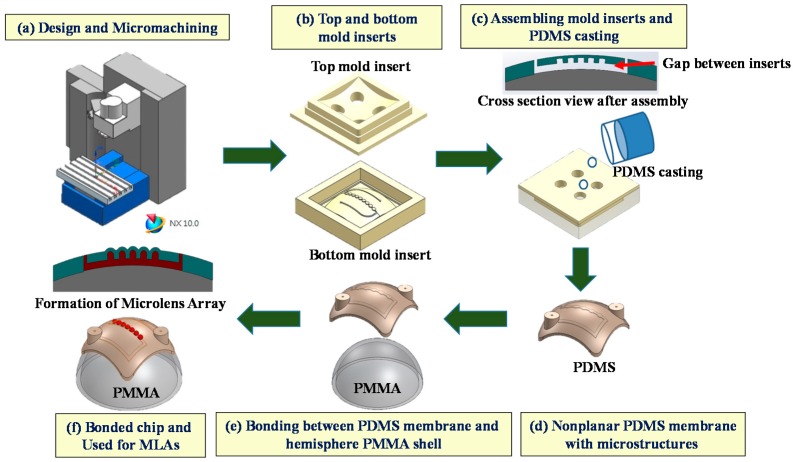
Microfabrication of a nonplanar poly(methylmethacrylate)/polymethylmethacrylate (PDMS/PMMA) microfluidic chip used for microlens array (MLA): (**a**) design and micromachining; (**b**) fabrication of the top and bottom nonplanar PMMA substrates via micromachining; (**c**) assembly of the top and bottom mold inserts to create a gap between substrates, followed by injection of PDMS to fabricate the nonplanar PDMS membrane; (**d**) a nonplanar PDMS membrane; (**e**) the heterogeneous solvent bonding used in the assembly of the nonplanar PDMS membrane on hemispherical PMMA substrate; (**f**) a bonded PDMS/PMMA chip used for MLAs.

After the micromilling techniques were adjusted for the polymeric nonplanar mold inserts and microfluidic chips, we implemented the proposed fabrication technique in conjunction with PDMS replication to create a nonplanar PDMS/PMMA chip, as shown in Figure 5. Micromilling was used to fabricate the top and bottom mold inserts (Figure 5b). Assembly of the inserts (Figure 5c) created a gap between the top and bottom mold inserts. PDMS casting was then used to create a nonplanar PDMS membrane (Figure 5d). Heterogeneous solvent bonding was used to bond the PDMS membrane on a hemispherical PMMA shell (Figure 5e) for use in a nonplanar microlens array (MLA) (Figure 5f) [23].

### 3.1. Micromachining of Polymeric Mold Inserts

**Figure 6:**
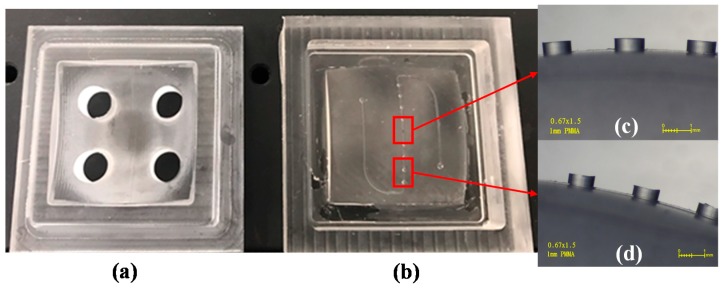
(**a**) A micromachined nonplanar top mold insert; (**b**) a micromachined nonplanar bottom substrate with tiny pillar structures; (**c**) the side view of the tiny pillars located in the central area of the nonplanar bottom substrate; (**d**) the side view of the tiny pillars located in the edge area of the nonplanar bottom substrate.

We fabricated the top and bottom mold inserts, as shown in Figure 6. Figure 6a shows the top mold insert with four through-holes, used in PDMS casting. Figure 6b shows the bottom mold insert with numerous tiny pillars and microchannels, for use in the creation of the PDMS membrane used for the MLA. Figure 6c,d present enlarged side-views of the tiny pillars on the bottom PMMA mold insert. Detailed machining process is listed in Tables S4 and S5.

### 3.2. PDMS Casting and Nonplanar Heterogeneous Bonding

**Figure 7:**
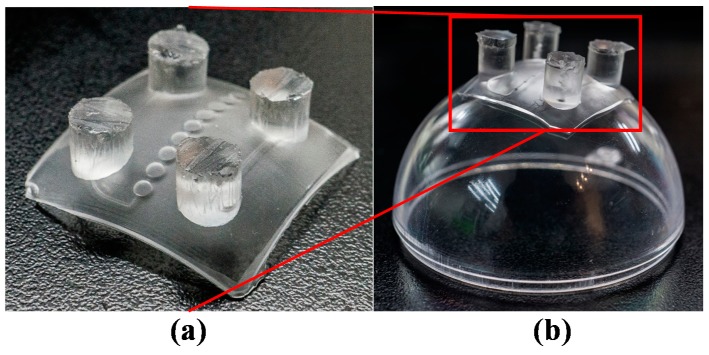
(**a**) Nonplanar-cast PDMS membrane; (**b**) PDMS membrane bonded on a hemispherical PMMA substrate.

The assembly of the top and bottom mold inserts created a gap between the two mold inserts (Figure 5c). PDMS casting was used to fabricate a nonplanar PDMS membrane within this gap (Figure 7a). Heterogeneous solvent bonding was used to bond the PDMS membrane to the hemispherical PMMA substrate, as described in a previous study [24]. Briefly, the steps were as follows: A plasma machine (PDC-32G, Harrick Plasma, Ithaca, NY, USA) was used to break the original polymer chains and create hydroxyl groups on the surfaces of the PMMA and PDMS. Modified PDMS was soaked in a 1% (*v*/*v*) aqueous solutions of aminosilane (ATPES) to create amine functionalities, whereas modified PMMA was soaked in a 1% (*v*/*v*) aqueous solutions of epoxysilane (GTPES) to create epoxy functionalities. The two substrates were then brought together to create an amine–epoxy bond. Figure 7b presents the bonded PDMS/PMMA chip used for MLAs.

**Figure 8:**
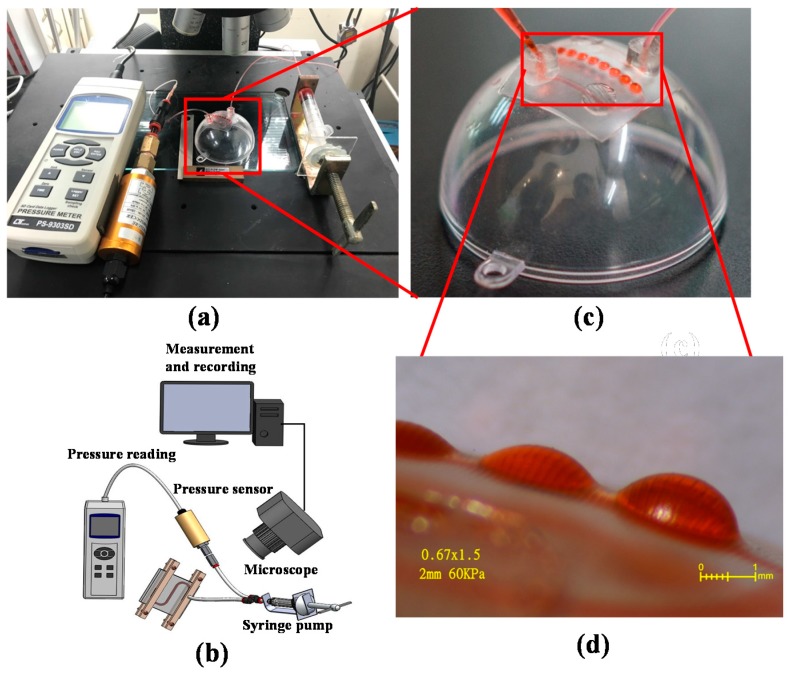
(**a**) The system used to deform the PDMS membrane to create the MLAs: pressure sensor, syringe pump, and microscope; (**b**) Schematic of the system; (**c**) MLAs on the hemispherical PMMA substrate; (**d**) the side view of the enlarged PDMS MLAs.

The system in Figure 8a was built to deform the PDMS membrane and form MLAs on the hemispherical PMMA substrate. The system included a sensor (PS100-10 Bar, Yalab, Taipei, Taiwan) to measure pressure within the microchannel, a manual syringe pump to push liquid into the microfluidic chip and to deform the PDMS membrane, and a microscope to record the deformation of the PDMS (Figure 8b). The key parameters to control the MLS was described in the previous article [23], and the diameter of each MLA in Figure 7a is 2 mm. 

## 4. Experiments and Discussion

### 4.1. Experiment Results: Nonplanar PMMA/PMMA Chip

**Figure 9:**
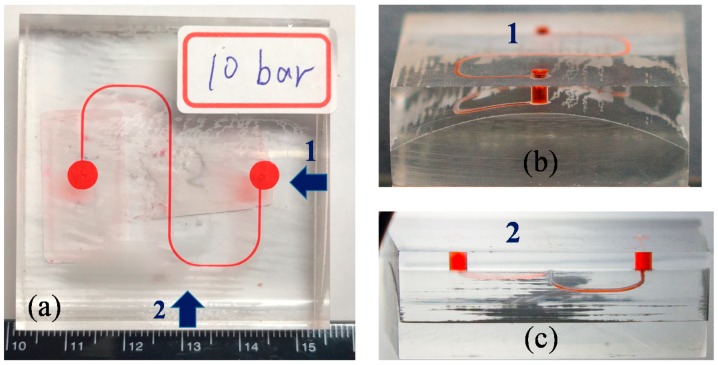
(**a**) Top view of the bonded nonplanar PMMA microfluidic chip with a S-microchannel; (**b**) the side view of the bonded nonplanar PMMA microfluidic chip from location 1; (**c**) the side view of the bonded nonplanar PMMA microfluidic chip from location 2.

**Figure 10:**
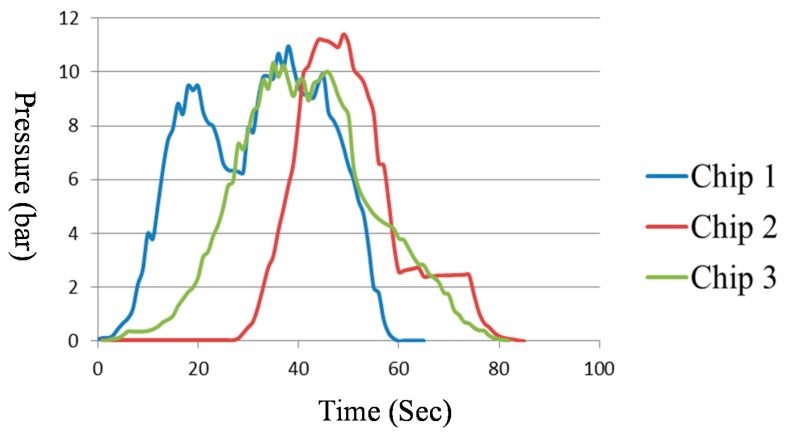
The experiment result of burst tests; three chips were tested and the results showed that the bonding strength remained intact beyond 10 bars.

The bonded PMMA/PMMA chip was subjected to several tests, including a leakage test and a burst test. Figure 9a shows a top-view image of the bonded PMMA/PMMA chip, indicating no signs of leakage. The side views in Figure 9b,c also revealed no signs of leakage. If inadequate parameters were used, then the surface contours of top and bottom substrates would not match and they would consequently cause a leakage (please check Supplementary Material Figures S1 and S2). In burst tests on three chips, the bonded chips remained intact beyond 10 bars, and the detailed experimental results are shown in Figure 10. From these results, we can conclude the following: (1) micromachined surface contours on the top PMMA substrates presented a good match with the contours on the bottom substrate, and (2) solvent bonding is applicable to the fabrication of nonplanar polymeric microfluidic chips.

### 4.2. Experiment Results: Nonplanar PDMS/PMMA Chip

Figure 8a presents the actual system used for the MLAs experiments. Figure 8b shows the PDMS MLAs on the hemispherical PMMA substrate. Figure 8c shows a side-view image of MLAs, wherein individual microlenses varied in sag height due to differences in the thickness of the membrane. With this PDMS MLAs, an artificial compound eye could be possibly realized and used for interesting application fields such as wavefront detectors or intelligent surveillance systems. Moreover, this experiment clearly demonstrated that micromachining can be used in conjunction with PDMS casting to create PDMS/PMMA microfluidic chips.

## 5. Conclusions

This paper reports a novel and efficient process for the fabrication of nonplanar 3D microfluidic chips. The crucial step involves the use of micromachining to fabricate nonplanar PMMA mold inserts. Several cutting parameters were adjusted to achieve smooth and well-defined surface contours. The efficacy of this fabrication process was demonstrated through the fabrication of a nonplanar PMMA/PMMA chip and a nonplanar PDMS/PMMA chip. An S-shaped microchannel was fabricated on the nonplanar PMMA substrate and sealed with another nonplanar PMMA cover via solvent bonding. Test results revealed no signs of leakage from the PMMA/PMMA chip and the bonding strength exceeded 10 bars. A PDMS membrane casted from two nonplanar PMMA substrates was bonded to the hemispherical PMMA substrate by solvent bonding method for use in MLAs. Red food dye injected into the microchannels under pressure deformed the PDMS membrane to create MLAs. These examples clearly demonstrate the efficacy of using micromachining to create nonplanar PDMS/PMMA microfluidic chips, as well as in the direct fabrication of PMMA/PMMA nonplanar microfluidic chips. These developments open the door to the fabrication of nonplanar microfluidic chips for applications requiring a 3D space for in vitro experiments.

## Supplementary Materials

The following are available online at http://www.mdpi.com/2072-666X/9/10/491/s1, Table S1: Machining parameters used for creating top PMMA nonplanar mold inserts (the figures shown here in are acquired from NX software and the colorbar shows the excess material left for machining. The left figure shows the tool path while the right figure shows the simulation, and the table below figures shows the machining parameters), Table S2: Machining parameters used for creating bottom PMMA nonplanar mold inserts (the figures shown here in are acquired from NX software and the colorbar shows the excess material left for machining. The left figure shows the tool path while the right figure shows the simulation, and the table below figures shows the machining parameters), Table S3: The measured surface roughness of the top and bottom substrates, and the measurement was done by a profilemeter (Hommel Werke T400 & P2000 Pick-up TKL 300, Jenoptik, Japan). Three chips were measured and nine points (from A~I) were measured, and the nine locations were shown in the Figure below the table, Table S4: Machining parameters used for creating top PMMA nonplanar mold inserts (the figures shown here in are acquired from NX software and the colorbar shows the excess material left for machining. The left figure shows the tool path while the right figure shows the simulation, and the table below figures shows the machining parameters), Table S5: Machining parameters used for creating bottom PMMA nonplanar mold inserts (the figures shown here in are acquired from NX software and the colorbar shows the excess material left for machining. The left figure shows the tool path while the right figure shows the simulation, and the table below figures shows the machining parameters), Figure S1: (a) the bonded microfluidic chip with leakage, due to the inadequate cutting parameters; (b) the enlarged figure of the leakage zone, Figure S2: (a) the machined nonplanar PMMA substrate with a square milling bit; (b) the machined nonplanar PMMA substrate with a ball milling bit.
