# Supplementary material for: Microfabrication of Nonplanar Polymeric Microfluidics
Source: Micromachines (Basel). 2018 Sep 25;9(10):491. doi: 10.3390/mi9100491 (PMC6215136; doi:10.3390/mi9100491)
Supplement: Supplementary file 1 [file micromachines-09-00491-s001.pdf]

Supplementary Materials: Microfabrication of Nonplanar Polymeric Microfluidics

Table S1. Machining parameters used for creating top PMMA nonplanar mold inserts (the figures shown here in are acquired from NX software and the colorbar shows the excess material left for machining. The left figure shows the tool path while the right figure shows the simulation, and the table below figures shows the machining parameters).

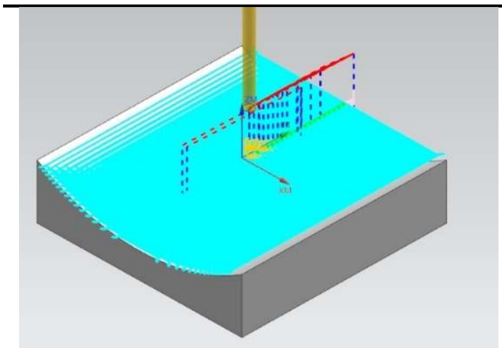

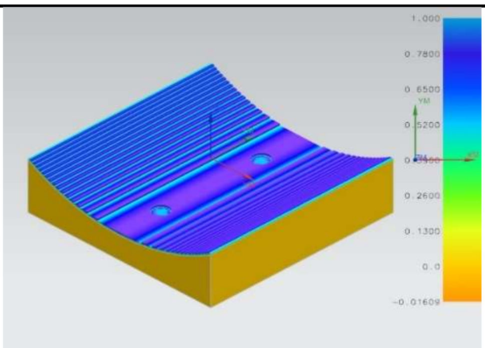

Step	Type	Diameter (mm)	Rotation Speed (rpm)	Depth of Cut (mm/min)	Stepover (%)
1	Square Milling	2.5 mm	7,000	700	50%

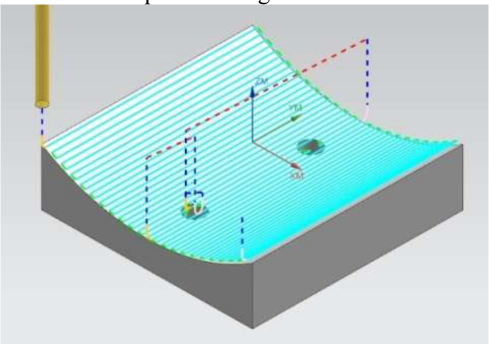

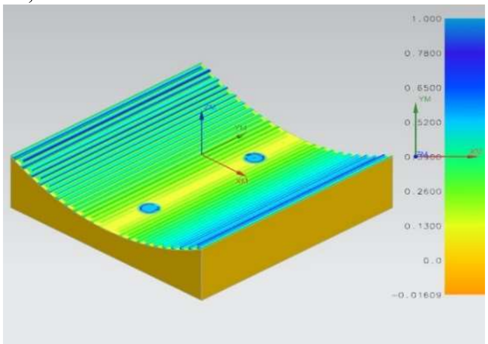

Step	Type	Diameter (mm)	Rotation Speed (rpm)	Depth of Cut (mm/min)	Stepover (%)
2	Ball Milling	1.5 mm	12,000	500	50%

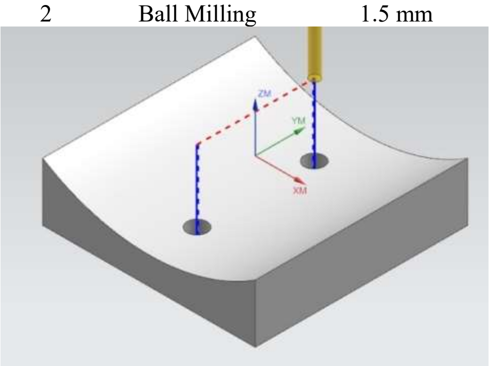

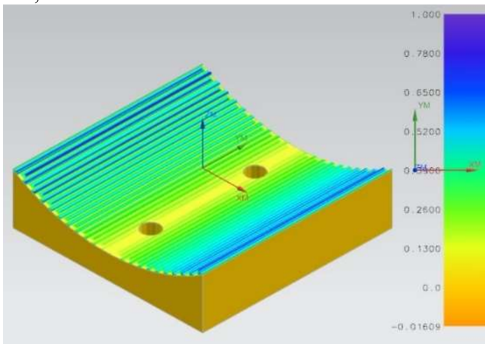

Step	Type	Diameter (mm)	Rotation Speed (rpm)	Depth of Cut (mm/min)	Stepover (%)
3	Square Milling	1 mm	11,000	650	75%

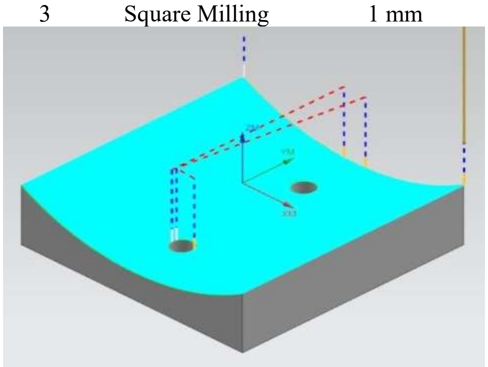

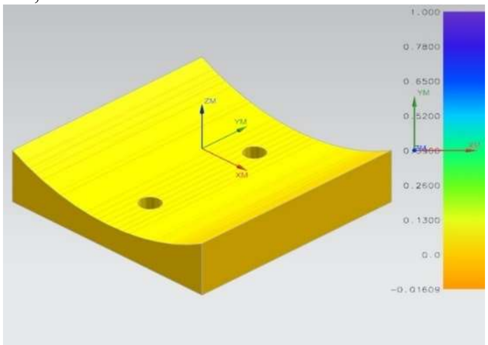

Step	Type	Diameter (mm)	Rotation Speed (rpm)	Depth of Cut (mm/min)	Stepover (%)
4	Ball Milling	0.5 mm	15,000	300	10%

Table S2. Machining parameters used for creating bottom PMMA nonplanar mold inserts (the figures shown here in are acquired from NX software and the colorbar shows the excess material left for machining. The left figure shows the tool path while the right figure shows the simulation, and the table below figures shows the machining parameters).

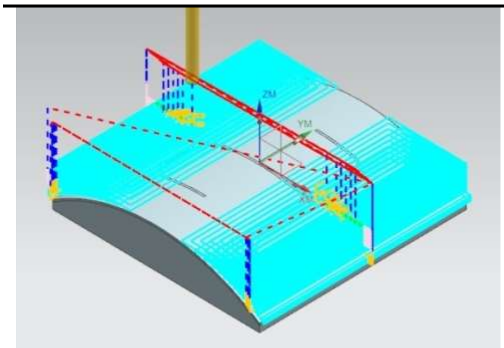

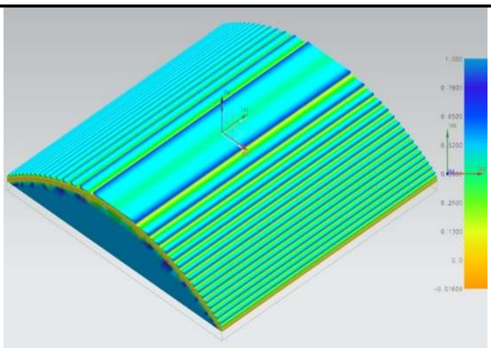

Step	Type	Diameter (mm)	Rotation Speed (rpm)	Depth of Cut (mm/min)	Stepover (%)
1	Square Milling	3 mm	7,000	800	50%

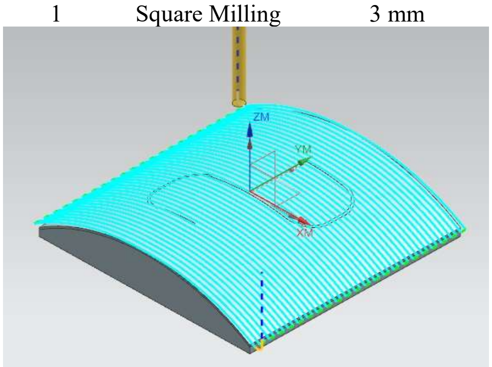

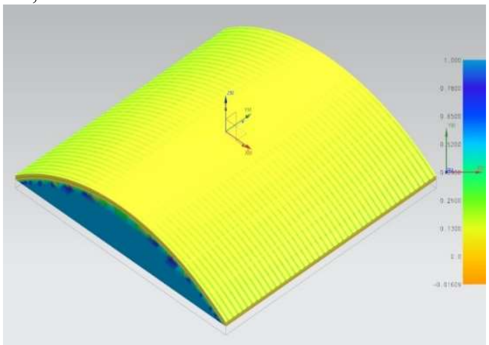

Step	Type	Diameter (mm)	Rotation Speed (rpm)	Depth of Cut (mm/min)	Stepover (%)
2	Ball Milling	1.5 mm	12,000	500	50%

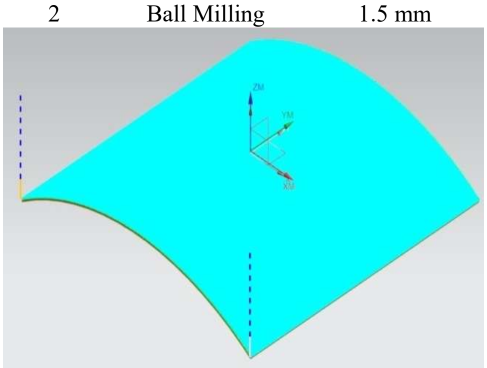

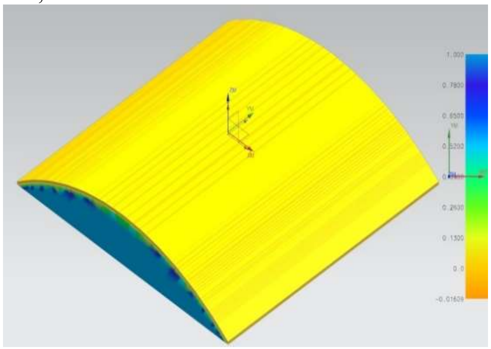

Step	Type	Diameter (mm)	Rotation Speed (rpm)	Depth of Cut (mm/min)	Stepover (%)
3	Ball Milling	0.5 mm	15,000	300	10%

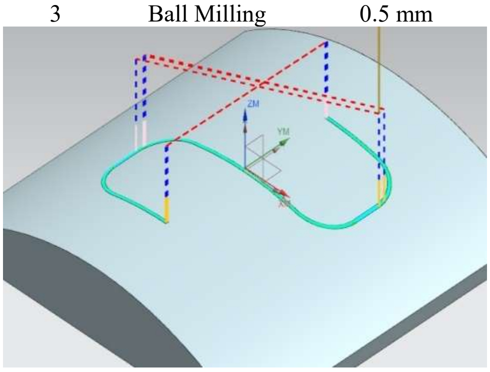

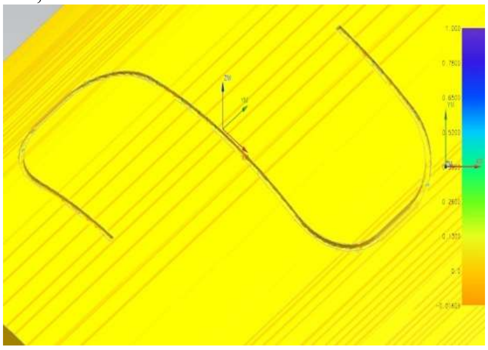

Step	Type	Diameter (mm)	Rotation Speed (rpm)	Depth of Cut (mm/min)	Stepover (%)
4	Ball Milling	0.3 mm	25,000	200	10%

Table S3. The measured surface roughness of the top and bottom substrates, and the measurement was done by a profilemeter (Hommel Werke T400 & P2000 Pick-up TKL 300, Jenoptik, Japan). Three chips were measured and nine points (from A~I) were measured, and the nine locations were shown in the Figure below the table.

Point		A	B	C	D	E	F	G	H	I	Average
Chip1	Bottom	1.13	0.97	0.95	0.68	0.68	0.72	1.15	1.16	1.11	**0.95**
	top	1.35	1.49	1.33	0.88	0.89	0.82	1.36	1.21	1.27	**1.17**
Chip2	Bottom	1.14	1.04	1.03	0.77	0.78	0.72	0.84	0.78	0.9	**0.89**
	top	1.16	1.15	1.07	0.93	0.94	0.89	1.5	1.36	1.26	**1.14**
Chip3	Bottom	1.09	1.05	0.97	0.65	0.78	0.74	0.89	0.9	0.86	**0.88**
	top	1.57	1.27	1.17	0.83	0.77	0.82	1.12	1.19	1.22	**1.10**

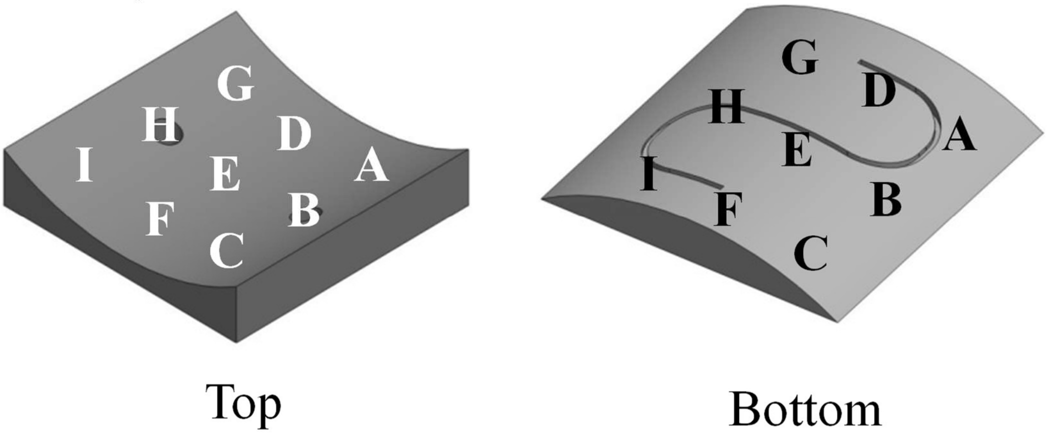

Table S4. Machining parameters used for creating top PMMA nonplanar mold inserts (the figures shown here in are acquired from NX software and the colorbar shows the excess material left for machining. The left figure shows the tool path while the right figure shows the simulation, and the table below figures shows the machining parameters).

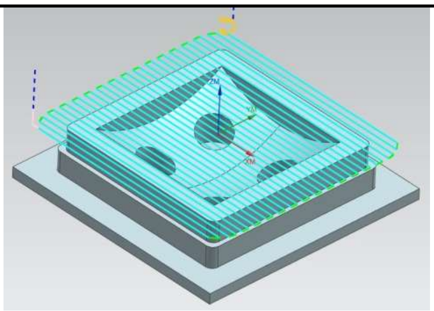

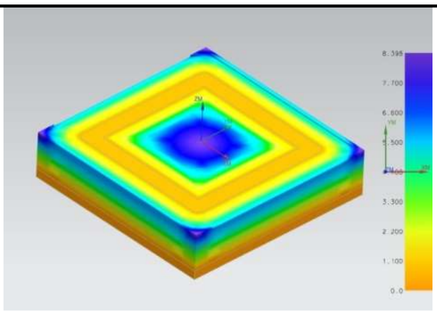

Step	Type	Diameter (mm)	Rotation Speed (rpm)	Depth of Cut (mm/min)	Stepover (%)
**1**	Square Milling	3 mm	7,000	800	75%

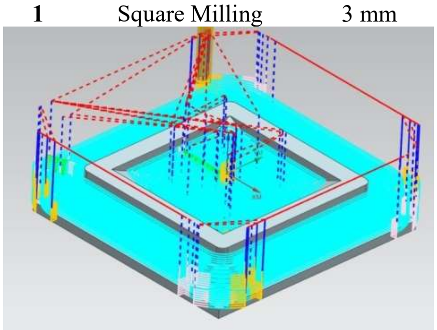

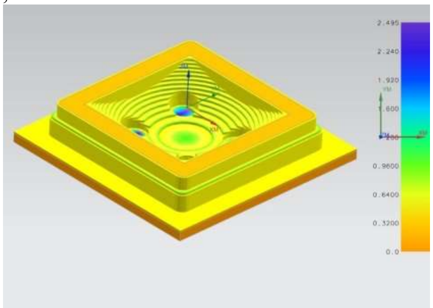

Step	Type	Diameter (mm)	Rotation Speed (rpm)	Depth of Cut (mm/min)	Stepover (%)
**2**	Square Milling	3 mm	7,000	800	75%

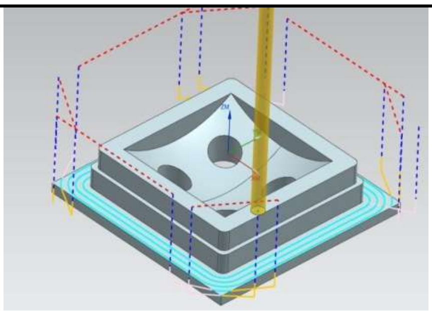
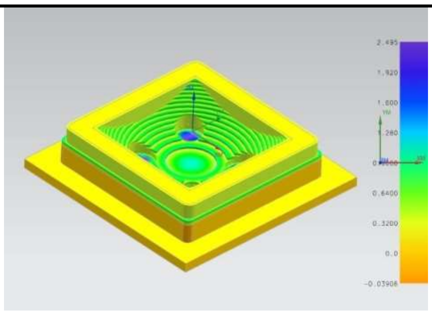

Step	Type	Diameter (mm)	Rotation Speed (rpm)	Depth of Cut (mm/min)	Stepover (%)
**3**	Square Milling	3 mm	7,000	800	75%

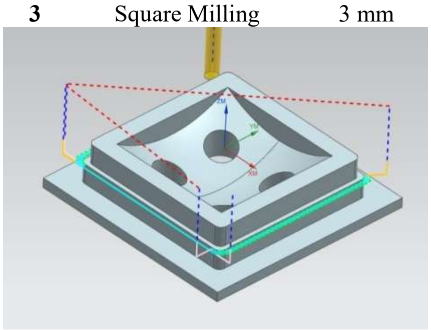
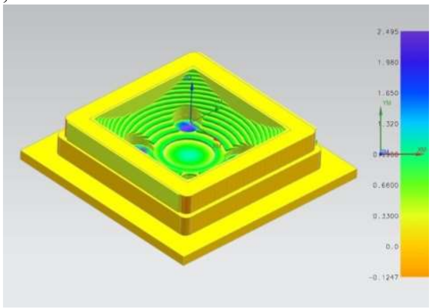

Step	Type	Diameter (mm)	Rotation Speed (rpm)	Depth of Cut (mm/min)	Stepover (%)
**4**	Square Milling	3 mm	7,000	800	75%

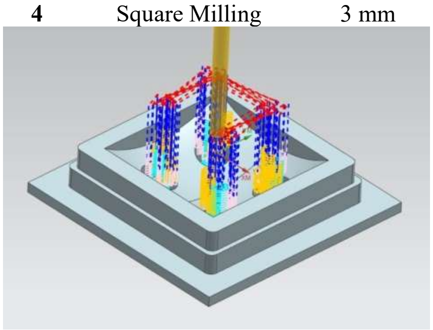
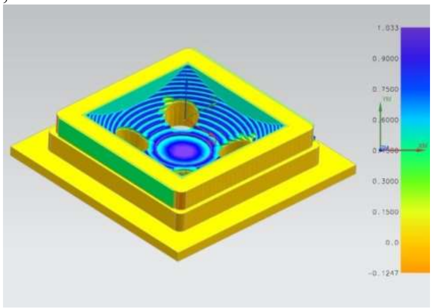

Step	Type	Diameter (mm)	Rotation Speed (rpm)	Depth of Cut (mm/min)	Stepover (%)
**5**	Square Milling	3 mm	7,000	800	50%

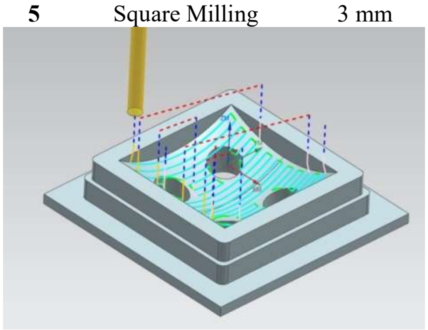
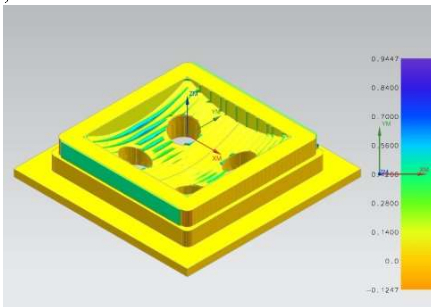

Step	Type	Diameter (mm)	Rotation Speed (rpm)	Depth of Cut (mm/min)	Stepover (%)
**6**	Ball Milling	1.5 mm	12,000	400	20%

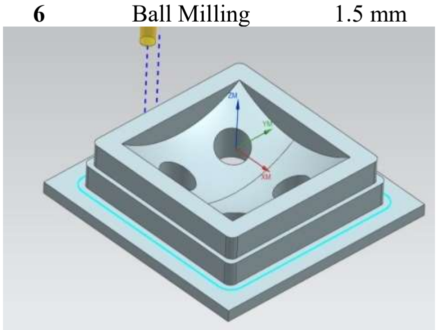
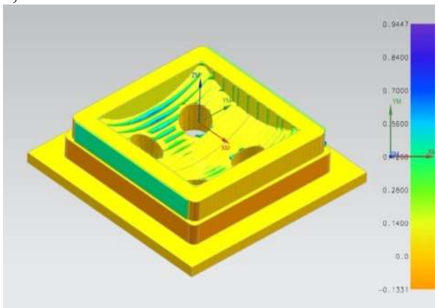

Step	Type	Diameter (mm)	Rotation Speed (rpm)	Depth of Cut (mm/min)	Stepover (%)
**7**	Square Milling	1 mm	11,000	600	30%

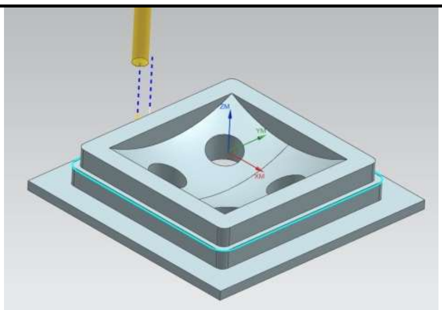

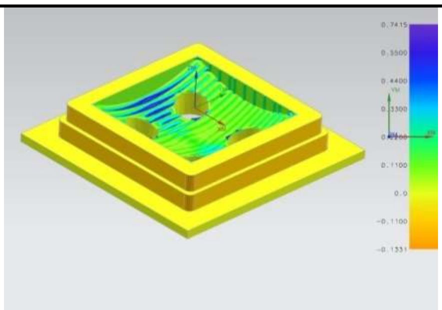

Step	Type	Diameter (mm)	Rotation Speed (rpm)	Depth of Cut (mm/min)	Stepover (%)
**8**	Square Milling	1 mm	11,000	600	30%

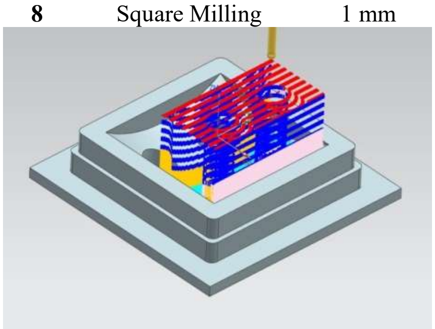

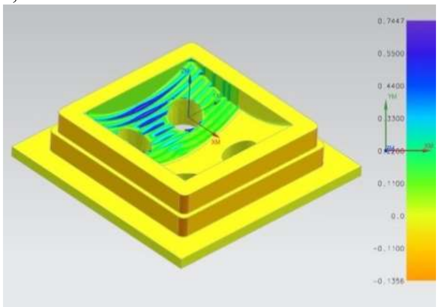

Step	Type	Diameter (mm)	Rotation Speed (rpm)	Depth of Cut (mm/min)	Stepover (%)
**9**	Square Milling	1 mm	11,000	600	30%

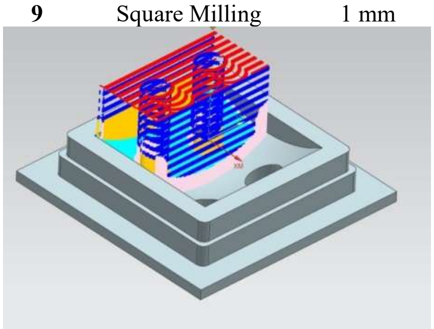

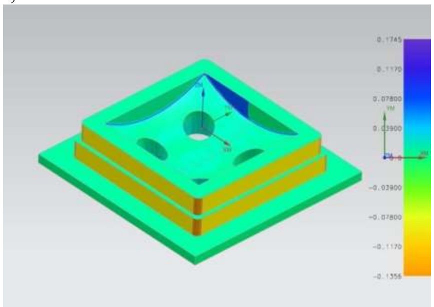

Step	Type	Diameter (mm)	Rotation Speed (rpm)	Depth of Cut (mm/min)	Stepover (%)
**10**	Square Milling	1 mm	11,000	600	30%

Table S5. Machining parameters used for creating bottom PMMA nonplanar mold inserts (the figures shown here in are acquired from NX software and the colorbar shows the excess material left for machining. The left figure shows the tool path while the right figure shows the simulation, and the table below figures shows the machining parameters).

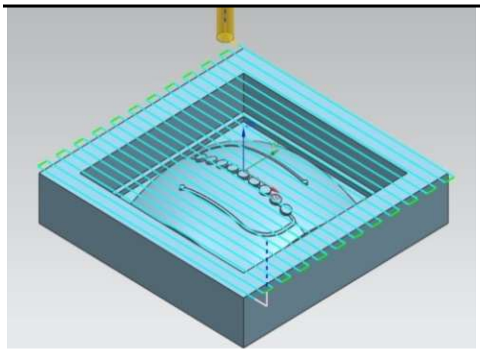

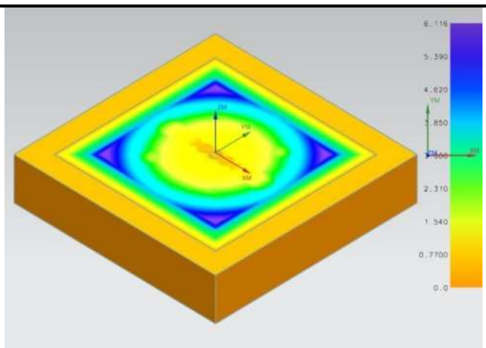

Step	Type	Diameter (mm)	Rotation Speed (rpm)	Depth of Cut (mm/min)	Stepover (%)
**1**	Square Milling	3 mm	7,000	800	75%

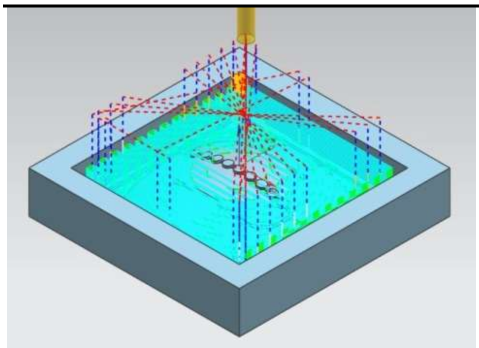
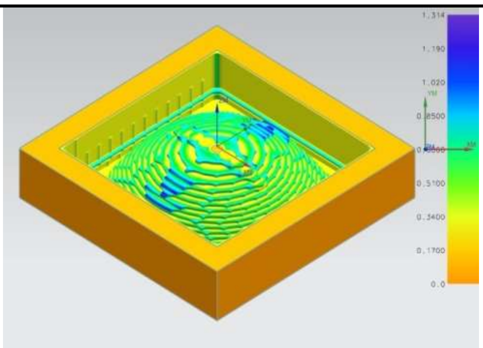

Step	Type	Diameter (mm)	Rotation Speed (rpm)	Depth of Cut (mm/min)	Stepover (%)
**2**	Square Milling	3 mm	7,000	800	50%

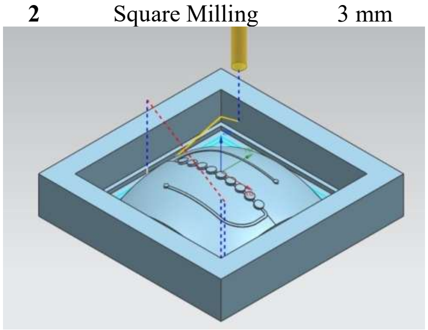
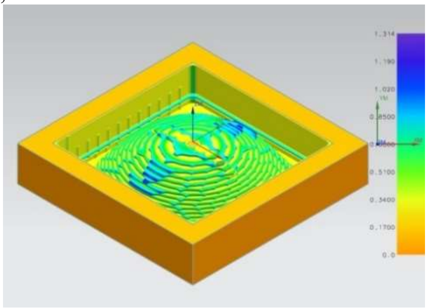

Step	Type	Diameter (mm)	Rotation Speed (rpm)	Depth of Cut (mm/min)	Stepover (%)
**3**	Square Milling	3 mm	7,000	700	20%

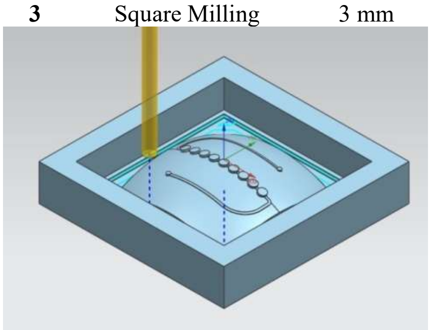
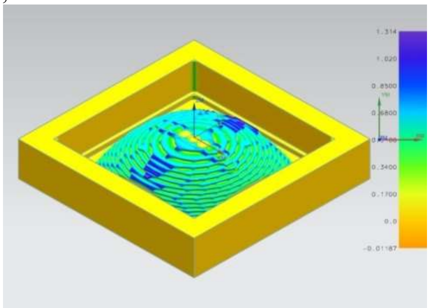

Step	Type	Diameter (mm)	Rotation Speed (rpm)	Depth of Cut (mm/min)	Stepover (%)
**4**	Square Milling	1 mm	11,000	600	20%

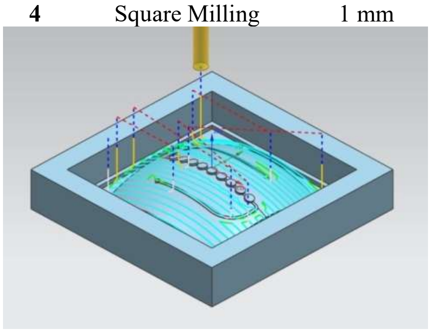
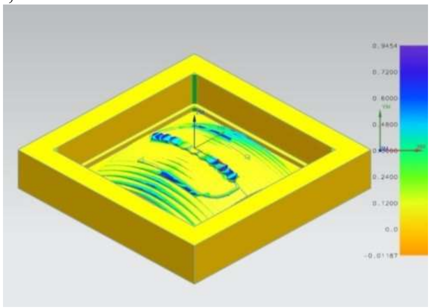

Step	Type	Diameter (mm)	Rotation Speed (rpm)	Depth of Cut (mm/min)	Stepover (%)
**5**	Ball Milling	1.5 mm	12,000	400	20%

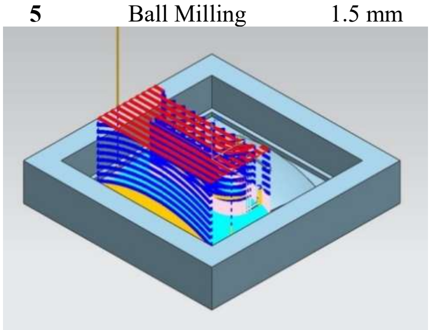
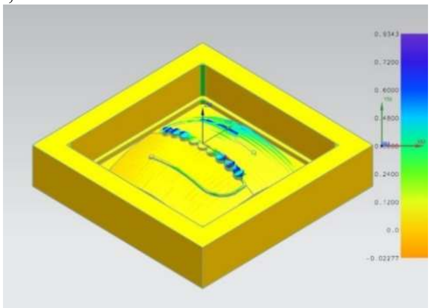

Step	Type	Diameter (mm)	Rotation Speed (rpm)	Depth of Cut (mm/min)	Stepover (%)
**6**	Ball Milling	0.5 mm	15,000	300	10%

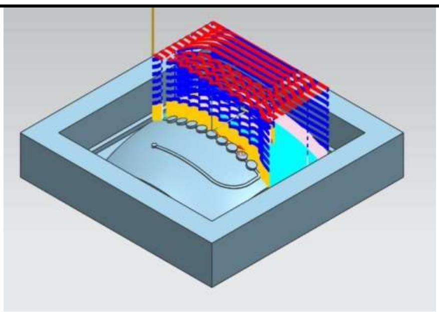
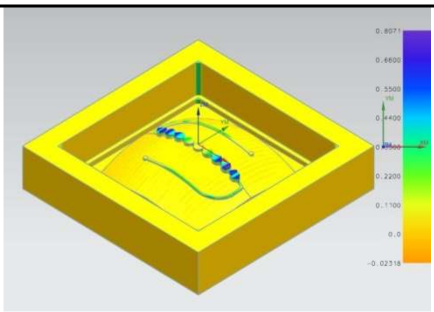

Step	Type	Diameter (mm)	Rotation Speed (rpm)	Depth of Cut (mm/min)	Stepover (%)
**7**	Ball Milling	0.5 mm	15,000	300	10%

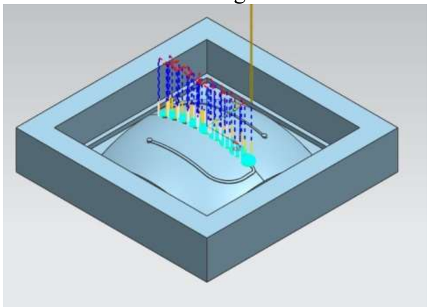
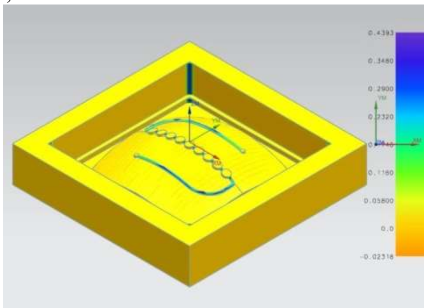

Step	Type	Diameter (mm)	Rotation Speed (rpm)	Depth of Cut (mm/min)	Stepover (%)
**8**	Square Milling	0.5 mm	15,000	300	10%

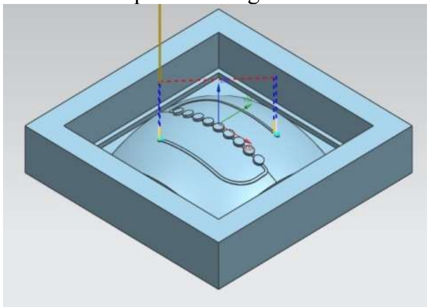
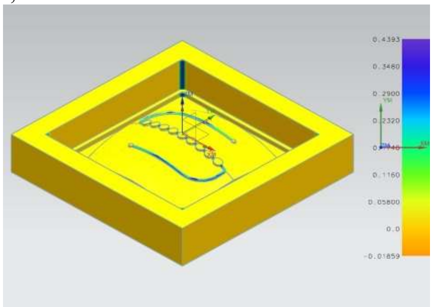

Step	Type	Diameter (mm)	Rotation Speed (rpm)	Depth of Cut (mm/min)	Stepover (%)
**9**	Square Milling	0.5 mm	15,000	300	10%

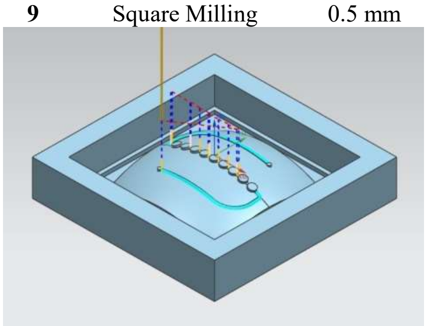
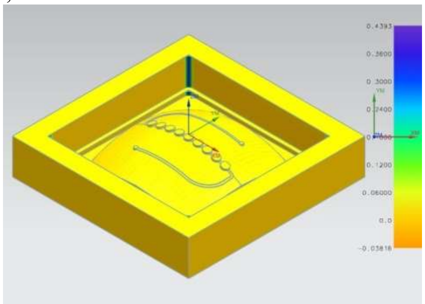

Step	Type	Diameter (mm)	Rotation Speed (rpm)	Depth of Cut (mm/min)	Stepover (%)
**10**	Square Milling	0.5 mm	15,000	300	10%

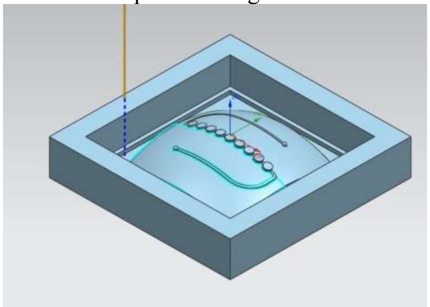
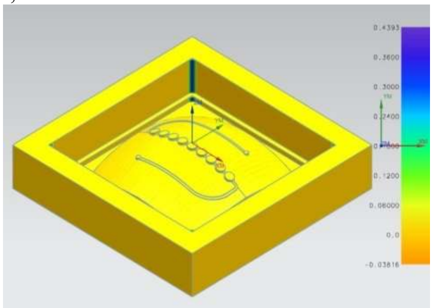

Step	Type	Diameter (mm)	Rotation Speed (rpm)	Depth of Cut (mm/min)	Stepover (%)
**11**	Square Milling	0.5 mm	15,000	300	10%

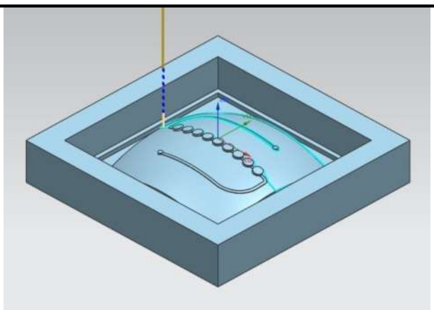

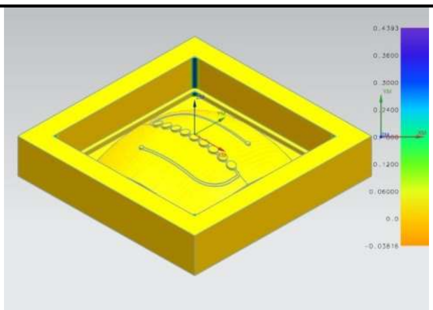

Step	Type	Diameter (mm)	Rotation Speed (rpm)	Depth of Cut (mm/min)	Stepover (%)
**12**	Square Milling	0.5 mm	15,000	300	10%

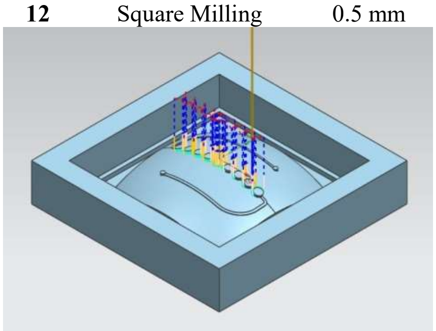

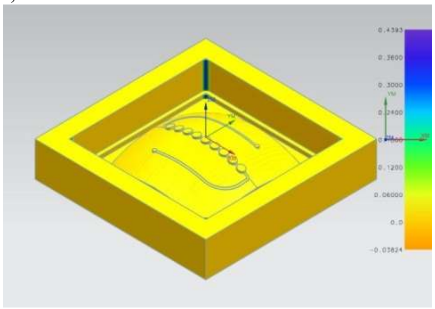

Step	Type	Diameter (mm)	Rotation Speed (rpm)	Depth of Cut (mm/min)	Stepover (%)
**13**	Square Milling	0.5 mm	15,000	300	10%

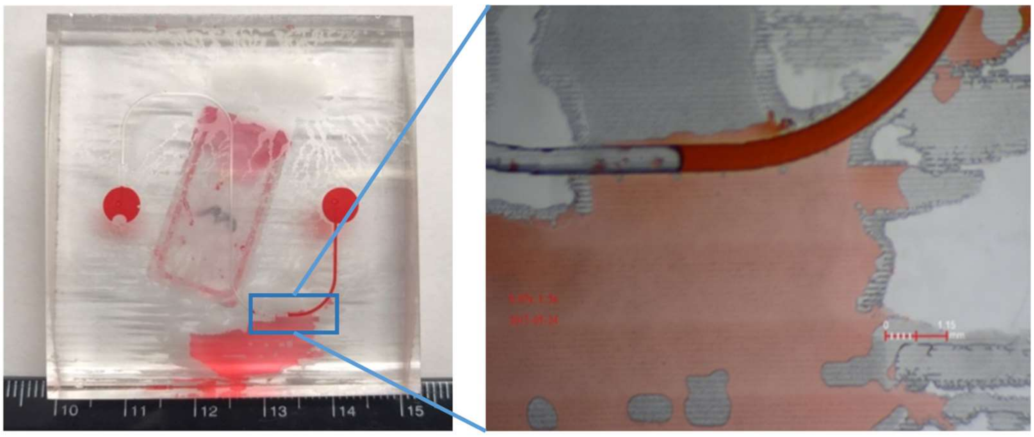

(a) **(b)**

Figure S1. (**a**) the bonded microfluidic chip with leakage, due to the inadequate cutting parameters; (**b**) the enlarged figure of the leakage zone.

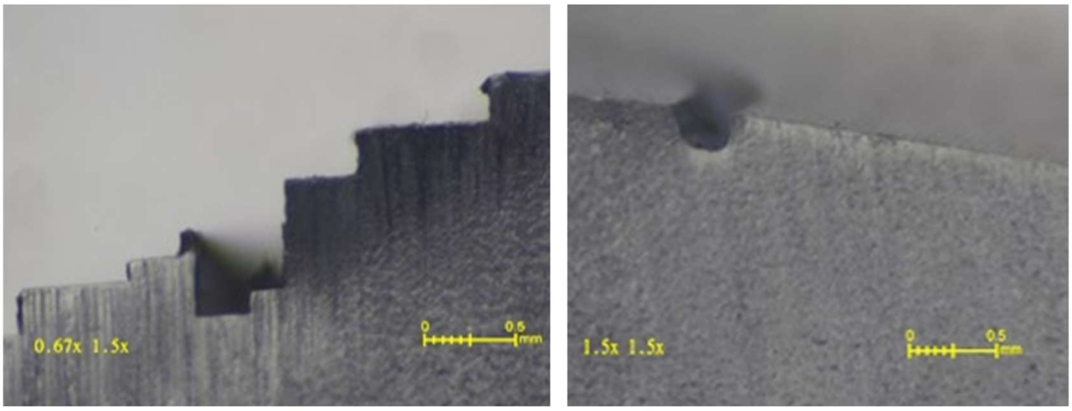

(a) **(b)**

Figure S2. (**a**) the machined nonplanar PMMA substrate with a square milling bit; (**b**) the machined nonplanar PMMA substrate with a ball milling bit.
